# Water-Borne Perovskite Quantum Dot-Loaded, Polystyrene Latex Ink

**DOI:** 10.3389/fchem.2018.00453

**Published:** 2018-10-23

**Authors:** Keke Huang, Lucheng Peng, Baijun Liu, Dongze Li, Qiang Ma, Mingyao Zhang, Renguo Xie, Dayang Wang, Wensheng Yang

**Affiliations:** ^1^State Key Laboratory of Inorganic Synthesis and Preparative Chemistry, College of Chemistry Jilin University, Changchun, China; ^2^Engineering Research Center of Synthetic Resin and Special Fiber, Ministry of Education, Changchun University of Technology, Changchun, China; ^3^China Star Optoelectronics Technology Co. Ltd, Shenzhen, China

**Keywords:** quantum dot, lead halide perovskites, luminescence, water-borne ink, polystyrene

## Abstract

Highly lipophilic nanocrystals (NCs) of cesium lead halides were successfully embedded in polystyrene (PS) particles by deliberately controlling the swelling of the PS particles in the mixtures of good and bad organic solvents. The resulting composite particles were readily transferred into water via simple stepwise solvent exchange, which yielded water-borne perovskite NC-based inks with outstanding structural and chemical stability in aqueous media. Minimal change in the photoluminescence (PL) of the NCs loaded in the PS particles was visible after 1 month of incubation of the composite particles in water in a broad pH range from 1 to 14, which could otherwise be hardly realized. Loading into the PS particles also made the NCs highly stable against polar organic solvents, such as ethanol, intense light irradiation, and heat. The NC PL intensity slightly changed after the composite particles were heated at 75°C and under irradiation of strong blue light (@365 nm) for 1 h. Furthermore, the PS matrices could effectively inhibit the exchange of halide anions between two differently sized perovskite NCs loaded therein, thereby offering a considerable technical advantage in the application of multiple perovskite NCs for multicolor display in the future.

## Introduction

In recent decades, a paradigmatic shift has occurred in inkjet printing, from conventional printing of newspapers and photographs to innovative printing of microelectronic circuits and rapid prototyping, for example, of tissue implants and many other niche applications (Singh et al., 2010). Consequently, the spectrum of inks has been largely extended to a diversity of functional nanomaterials, such as metallic nanoparticles (Liang et al., 2016), semiconductor quantum dots (QDs) (Kim et al., 2011; Carey et al., 2015), and carbon nanotubes (Wang et al., 2012), to translate their peculiar functions into technological advances. In parallel, the awareness of environmental friendly manufacturing is prospering at present, and hence considerable attention is being given to the development of water-borne ink formulations. However, in most cases, high-quality nanomaterials are produced in organic media (Murray et al., 2000; Weller, 2003), and their designated functions are deteriorated after phase transfer into water to a considerable extent (Medintz et al., 2005; Jamieson et al., 2007). In this context, the present work aims to develop water-borne inks from perovskite QDs of CsPbX_3_ (X = Cl, Br, and I) by taking advantage of their outstanding optical properties (Protesescu et al., 2015; Swarnkar et al., 2015; Tong et al., 2016; Liu et al., 2017), which, however, remains a daunting challenge as most of the currently available perovskite QDs readily incur severe degradation upon contact with water (Frost et al., 2014). Furthermore, the anion-exchange reaction between different halide QD particles when used together remain a major issue that obstructs their practical applications (Akkerman et al., 2015; Nedelcu et al., 2015). To circumvent the challenge, we successfully incorporated preformed CsPbX_3_ QDs into polystyrene (PS) latex particles via stepwise solvent exchange methodology. The resulting CsPbX_3_ QD-loaded PS particles were able to disperse in aqueous media with outstanding chemical and structural stability. The photoluminescence (PL) behavior of the resulting composite particles in water is fairly comparable with the original QD dispersions in organic media. Anion exchange between the loaded QDs with different compositions was slightly visible.

Since the seminal work of Protesescu et al. (2015), CsPbX_3_ QDs have rapidly emerged as promising candidates for optoelectronic applications, such as light-emitting diodes (LED), lasing, photodetection, and backlight display (Wang et al., 2015; Amgar et al., 2016; Li G. et al., 2016; Ling et al., 2016; Li X. et al., 2016; Palazon et al., 2016b; Rainò et al., 2016; Ramasamy et al., 2016; Swarnkar et al., 2016; Xu et al., 2016; Zhang et al., 2016; Li J. H. et al., 2017; Zhou et al., 2017) because of their high PL quantum yields (50–90%), extremely narrow full-width at half-maximum, and broad emission spectral tunability in visible-light region.(Protesescu et al., 2015) Nevertheless, the success of CsPbX_3_ QDs in technical applications is largely bottlenecked by their high vulnerability to polar solvents, humidity, light, and temperature of the surrounding environment (Frost et al., 2014; Huang et al., 2016a; Wang et al., 2016a), in response to which the PL behavior of the perovskite QDs was severely deteriorated as a result of the QD degradation. To address this challenge, a number of efforts have been devoted to deliberately adjust the stabilizing ligands or the perovskite compositions to reduce the environmental vulnerability of the QDs derived thereof (Dirin et al., 2016; Huang et al., 2016; Palazon et al., 2016a; Sun et al., 2016; Wang et al., 2016b; Li Z. et al., 2017). Although the stability of QDs has been significantly improved, as-prepared QDs modified by inorganic materials or organic small molecules still show poor stability in polar solvent. Recently, polymer matrices have been utilized for *in situ* growth of perovskite QDs therein, leading to QD/polymer nanocomposites with substantially improved stability against water, heat, and light (Huang et al., 2016a,b; Meyns et al., 2016; Raja et al., 2016; Wang et al., 2016a; Hai et al., 2017). However, the preparation of QD–polymer composites by *in situ* growth or direct incorporation strategy cannot inhibit dynamic exchanges of anions between the growing perovskite QDs. This condition fails to incorporate the QDs with different compositions in polymer matrices. One of the merits of perovskite QDs is their flexibility to tune their PL characteristics based on the composition.

## Materials and methods

### Chemicals

Technical grade Octadecene (ODE, 90%) and Oleic acid (OA, 90%) were purchased from Alfa Aesar. Cesium carbonate (Cs_2_CO_3_, 99.9%), lead chloride (PbCl_2_, 99.999%), lead bromide (PbBr_2_, 99.999%), lead iodide (PbI_2_, 99%), and Oleylamine (OLA, 70%) were purchased from Aldrich. Hexane and toluene were obtained from Beijing Chemical Reagent Ltd., China. All reagents were used as received without further experimental purification.

### Preparation of Cs-oleate precursor

Cs_2_CO_3_ (0.652 g, 2 mmol), OA (2.5 mL, 7.5 mmol), and ODE (17.5 mL) were added into a 50 mL three-necked flask, dried for 1 h at 120°C, and then heated under N_2_ to 150°C until Cs_2_CO_3_ reacted with OA. Since Cs-oleate precipitates out of ODE at room temperature, it must be preheated to 100°C before use.

### Preparation of PbX_2_ stock solution

Lead halide (0.2 mmol), Oleic acid (0.5 mL), and Oleylamine (0.5 mL) (Trioctylphosphine (0.5 mL) for lead chloride) were mixed with ODE (4.0 mL) in a 50 mL three-necked flask. The reaction mixtures were degassed under vacuum for 30 min and purged with argon. The flask was heated to 150°C until a clear solution was formed, and then cooled to the room temperature (25°C).

### Synthesis of CsPbBr_3_ nanoparticles

In a typical synthesis of CsPbBr_3_ nanocubes, ODE (5 mL) and PbBr_2_ (0.2 mmol) were loaded into a 25 mL three-necked flask and dried under vacuum for 1 h at 120°C. Dried oleylamine (0.5 mL, OLA) and dried OA (0.5 mL) were injected at 120°C under N_2_. After complete solubilization of a PbBr_2_ salt, the temperature was raised to 170°C and Cs-oleate solution (0.2 mL, 0.2 M in ODE, prepared as described above) was quickly injected and 5 s later the reaction mixture was cooled in an ice-water bath. The aggregated nanocubes were then separated by centrifuging at 10,000 rpm for 10 min.

### Anion-exchange reaction

Anion-exchange Reaction could happen between Cl-Br, Br-Cl, Br-I, and I-Br, and the PbX_2_ stock solution was the anion source. In a typical anion-exchange reaction, such as CsPbBr_3_ to CsPb(Br/I)_3_, the whole reaction process was performed at room temperature with a certain amount of purified CsPbBr_3_ nanocubes dispersed in toluene, followed by the drop-wise addition of PbI_2_ stock solution to the sample, and after about 3 min, the reaction was completed.

### Preparation of polystyrene (PS) microspheres

The preparation of mono-disperse, highly cross-linked, and re-dispersible polystyrene microspheres was carried out by seed swelling method. A typical seed swelling process for the preparation of highly cross-linked polystyrene particles with the size of several micorometer involves the following steps: 0.3 g of polystyrene seed particles (400 nm) and 100 g of SDS (0.25 wt% for water) were added into a three-necked round-bottom flask and subjected to ultrasonic dispersion (about 30 min). Then, 1 g cyclohexane was ultrasonically dispersed with 50 g SDS (0.25 wt% for water), wherein the dispersion time here was also 30 min. The dispersion of cyclohexane was placed in the bottom and the swelling of polystyrene seed was carried out as the first step. The swelling temperature was 30°C and the swelling time was 10 h. After the first step of swelling, the system that 10 g styrene monomer, 5 g crosslinking agent vinyl benzene (DVB), 0.3 g initiator benzoyl peroxide (BPO) were disperse by 100 g SDS solution (0.25 wt%) under ultrasonic conditions and the dispersion time was 60 min. Afterwards, the system was added into the flask to carry out the second seed-swelling at 30°C for 8 h. After performing the swelling twice, 80 g polyvinyl alcohol solution at a concentration of 2.5 wt% and 0.02 g CuCl_2_ were added to the flask, and the temperature was increased to 85°C with a reaction time of 12 h. Finally, the obtained slurry was centrifuged at 5,000 rpm for 10 min, and washed with ethanol, followed by washing with water for three times. The product was dried at 50°C under vacuum for 24 h.

### Preparation of the NCs-PS particles

The polystyrene particle dispersions (3 mL, 1^*^10^−11^ mol) were added into a 15 mL centrifugation tube. After centrifugation, the upper supernatant was decanted and the particle sediment was dispersed using ethanol. This centrifugation/decanting/re-dispersion cycle was repeated for three times and then the polystyrene particles were transferred from water into ethanol. Following the same procedure, the polystyrene particles were subsequently transferred from ethanol into hexane, thereby yielding the PS particle dispersion in hexane. Next, the dispersions of the PS in hexane (10 mL) were mixed with the dispersions of perovskite NCs in toluene (1 mL, 2^*^10^−8^ mol). After stirring for 2 h, the NCs-PS particles were separated from the organic media by centrifugation. The NCs-PS sample was then purified three times by centrifugation and re-dispersed into water for characterization.

### Photooxidation during UV light irradiation

The photooxidation measurements of the colloidal perovskite NCs solution and NCs-PSP solution were analyzed using two bottom-transparent, airtight, quartz colorimetric cuvettes. At periodic intervals, the PL spectra of the colloidal perovskite NCs solution and NCs-PSP solution were recorded after illuminating with 365 nm LED light (8 Watt, 3UV^TM^-38 UV Lamp, China).

## Characterizations

TEM observations were made using a JEOL 100CX transmission electron microscope with an acceleration voltage of 200 kV. Carbon-coated copper grids were dipped in the hexane solutions to deposit the NCs onto the films. The luminescent quantum efficiency was determined using an integrating sphere (150 mm diameter, BaSO4 coating) from the Edinburg FLS920 phosphor meter. Quantum yield is defined as the integrated intensity of the luminescence signal divided by the integrated intensity of the absorption signal. Absorption intensity was calculated by subtracting the integrated intensity of the light source with the sample in the integrating sphere from that of the light source with a blank sample in the integrating sphere. Dynamic light scattering (DLS) measurements were performed (based on the number of particles) using a particle size analyzer (BI-90 Plus, Brookhaven Instruments) with a scattering angle of 90°. UV-visible spectra were recorded on an HP8453 UV-visible spectrophotometer. PL spectra were recorded on an Edinburgh FLS920 spectrophotometer. Thermogravimetric analysis (TGA) measurements were performed using Pyris (PerkinElmer, Inc). The sample was purified for further use by centrifugation (Shanghai Luxiangyi centrifuge instrument TG16-WS). The lead ions from water were measured by means of inductively coupled plasma atomic emission spectroscopy (ICP-AES, Thermo Elemental IRIS1000). It should be noted that the sample was purified three times by centrifugation and redispersion in water. The detection limit was 17.8 ng/mL in our experiment.

### Thermogravimetric analysis (TGA)

Thermogravimetric analysis (TGA) was used to detect the amount of CsPbBr_3_ nanocubes in a PSP. After purification, the sample was dried in a vacuum oven overnight at 50°C under reduced pressure (30 in. Hg), and the resulting sample was then scanned from 100 to 700°C with a heating rate of 5°C/min under N_2_ atmosphere (Figure S12). Total mass of the sample was 10.344 mg before TGA and the residual mass was 0.036 mg after TGA. Hence, we assume that the mass of CsPbBr_3_ nanocubes was 0.036 mg and the mass of PSP was 10.308 mg. The calculation was performed as follows:

(S1) $$\begin{array}{r} V\;total\;psp=\frac{m\;total\;psp}{\rho psp} \end{array}$$

(S2) $$\begin{array}{r} V\;total\;NCs=\frac{m\;total\;NCs}{\rho NCs} \end{array}$$

In Equations S1, S2, ρ_psp_ is 1.05 g/cm^3^ and ρ_NCs_ is 4.94 g/cm^3^.

(S3) $$\begin{array}{r} V\;psp=\frac{4}{3}\pi R_{psp}^{3} \end{array}$$

(S4) $$\begin{array}{r} V\;NCs=\frac{4}{3}\pi R_{NCs}^{3} \end{array}$$

In Equations S3, S4, R_psp_ is 1 μm and R_NCs_ is 8.4 nm.

(S5) $$\begin{array}{r} N\;PSP=\frac{V\;total\;PSP}{V_{PSP}} \end{array}$$

(S6) $$\begin{array}{r} N\;NCs=\frac{V\;total\;NCs}{V_{NCs}} \end{array}$$

(S7) $$\begin{array}{r} N=\frac{N\;NCs}{N_{PSP}} \end{array}$$

The Equations S5, S6 could be determined using Equations S1–S4. According to the Equation S7, we could calculate that there are nearly 640 CsPbBr_3_ nanocubes in one PS particle by assuming no liangds in polystyrene particles and the organic stabilizing ligands 700°C. It should be noted that the final mass for pure PS particles was almost zero after 700°C (Figure S13). Therefore, the final mass should be attributed to the incorporated NCs in PS particles.

## Results and discussion

**Figure 1:**
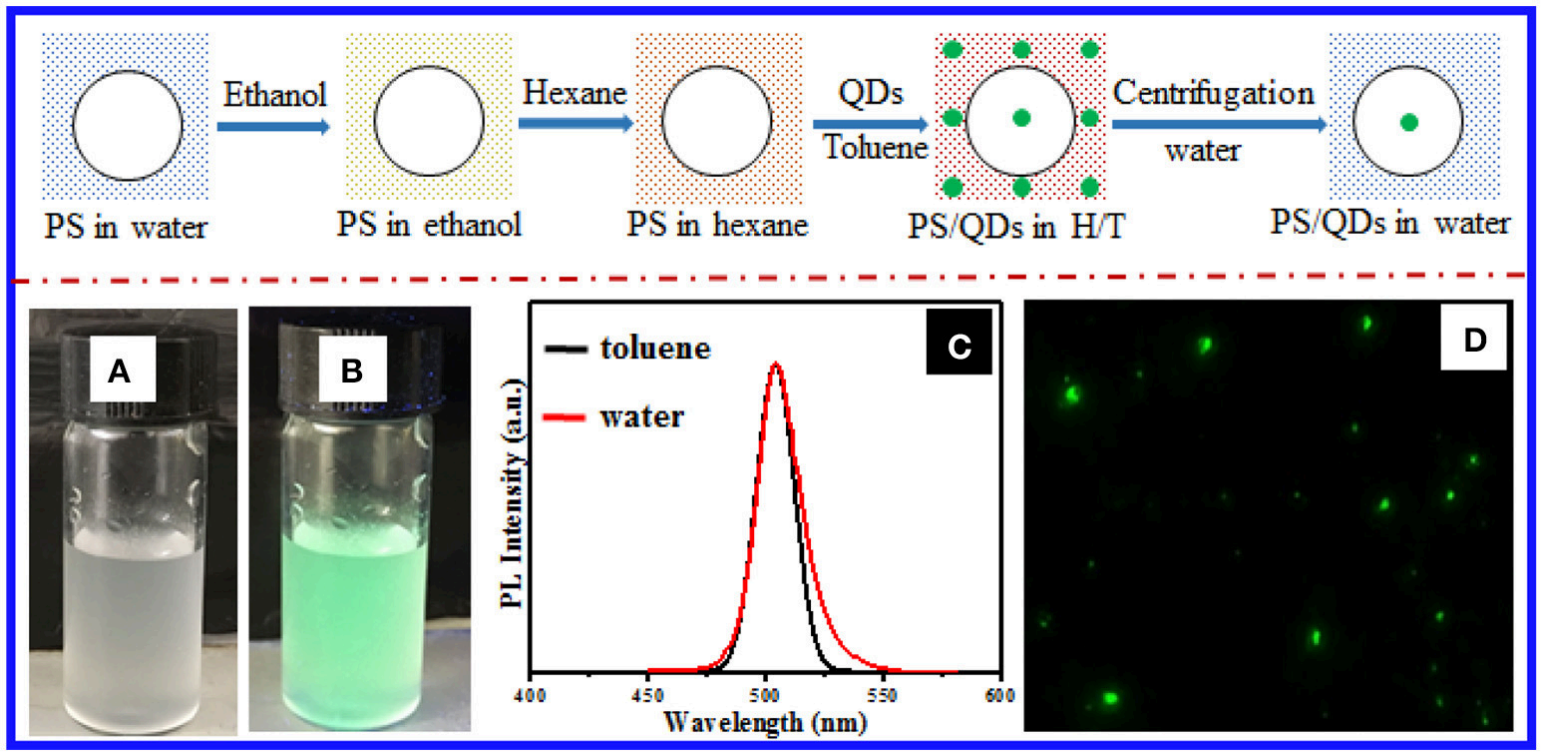
(Top) Schematic of incorporating lipophilic perovskite NCs into the PS particles via solvent exchange, where the large circle represents the PS particles and the green dots signify the NCs. Photographs of the aqueous dispersions of composite particles bearing 11 nm CsPbBr_3_ NCs loaded into 1 μm PS particles, shot **(A)** under sunlight and **(B)** UV irradiation (λ = 365 nm). **(C)** Emission spectra of the toluene dispersions of 11 nm CsPbBr_3_ NCs (black curve) and the aqueous dispersions of the CsPbBr_3_ NC-loaded PS composite particles. **(D)** Fluorescence micrograph of the CsPbBr_3_ NC-loaded PS composite particles.

In the present work, we established a stepwise solvent exchange methodology to incorporate the prepared perovskite NCs into PS latex particles directly (Figure 1A and Experimental details in Supporting Information). On the basis of our previous success in loading hydrophobic CdSe NCs into hydrogel beads (Bai et al., 2010), we first transferred the commercially available or prepared PS particles from water to the dispersions of lipophilic CsPbX_3_ NCs on hexane, which is a poor solvent for PS, via solvent exchange with the aid of ethanol as the intermediate solvent. Second, we added a small volume of toluene to the resulting hexane dispersions to deliberately stimulate the PS particles to swell and thus imbibe the NCs into the PS matrices, thereby yielding CsPbX_3_ NC-loaded PS particles. Third, we collected the resulting composite particles via centrifugation and directly dispersed them in water after consecutively decanting the hexane/toluene supernatants and removing the residual organic solvents via evaporation in air. Figure 1B shows that the composite particles, which were derived from 11 nm CsPbBr_3_ NCs (Figure S1) embedded in 1 μm PS particles (Figure S2), could well disperse in water and show strong green emission under irradiation of a UV lamp. As shown in Figure 1C, the green fluorescence came from the composite particles rather than the surrounding media, which showed the successful confinement of the CsPbBr_3_ NCs in the particles. The loading efficiency of NCs into the PS particles was roughly estimated by thermogravimetric analysis to be 640 particles in one PS particle (see Experimental section). Notably, calculating the quantum yield of loaded NC in particles was difficult due to strong scattering from absorption spectra of samples. The average sizes and size distribution of the resulting composite particles slightly changed after 5 days on the basis of dynamic light scattering analysis, thereby indicating good stability of the composite particles in water (Figure S3). The emission spectra of the CsPbBr_3_ NC-loaded PS particles in water were almost identical to those of the original dispersions of the CsPbBr_3_ NCs in toluene (Figure 1C), which indicated minimal change in the NC sizes after loading into the PS particles and transferring into water. Figure 1D shows the NC-loaded PS particles after careful size selection under UV irradiation. Notably, the large aggregation from the composite particles during the solvent exchange and storage was removed by centrifugation, although the green emission from large composite particles was still observable in water, as shown Figure S4. In the current work, the optimal hexane-to-toluene (H/T) volume ratio in the organic dispersions of CsPbBr_3_ NC-loaded PS particle mixtures was found to be ~10:1, at which the PS particles properly swelled so they could efficiently imbibe the CsPbBr_3_ NCs from the surrounding and simultaneously retain the structural integrity (Figure S5B). Otherwise, the PS particles incurred noticeable decomposition at low H/T volume ratio (pure toluene; Figure S5A) or almost no NCs were loaded into the PS particles at high H/T volume ratio (pure hexane; Figure S5C). After transfer of the composite particles from water back into pure toluene solvent to liberate the CsPbBr_3_ NCs, the NC size and morphology were almost identical to those of the original ones (Figure S6). This result was additional proof that the NC structures remained intact during loading into the PS particles and the subsequent phase transfer to water.

**Figure 2:**
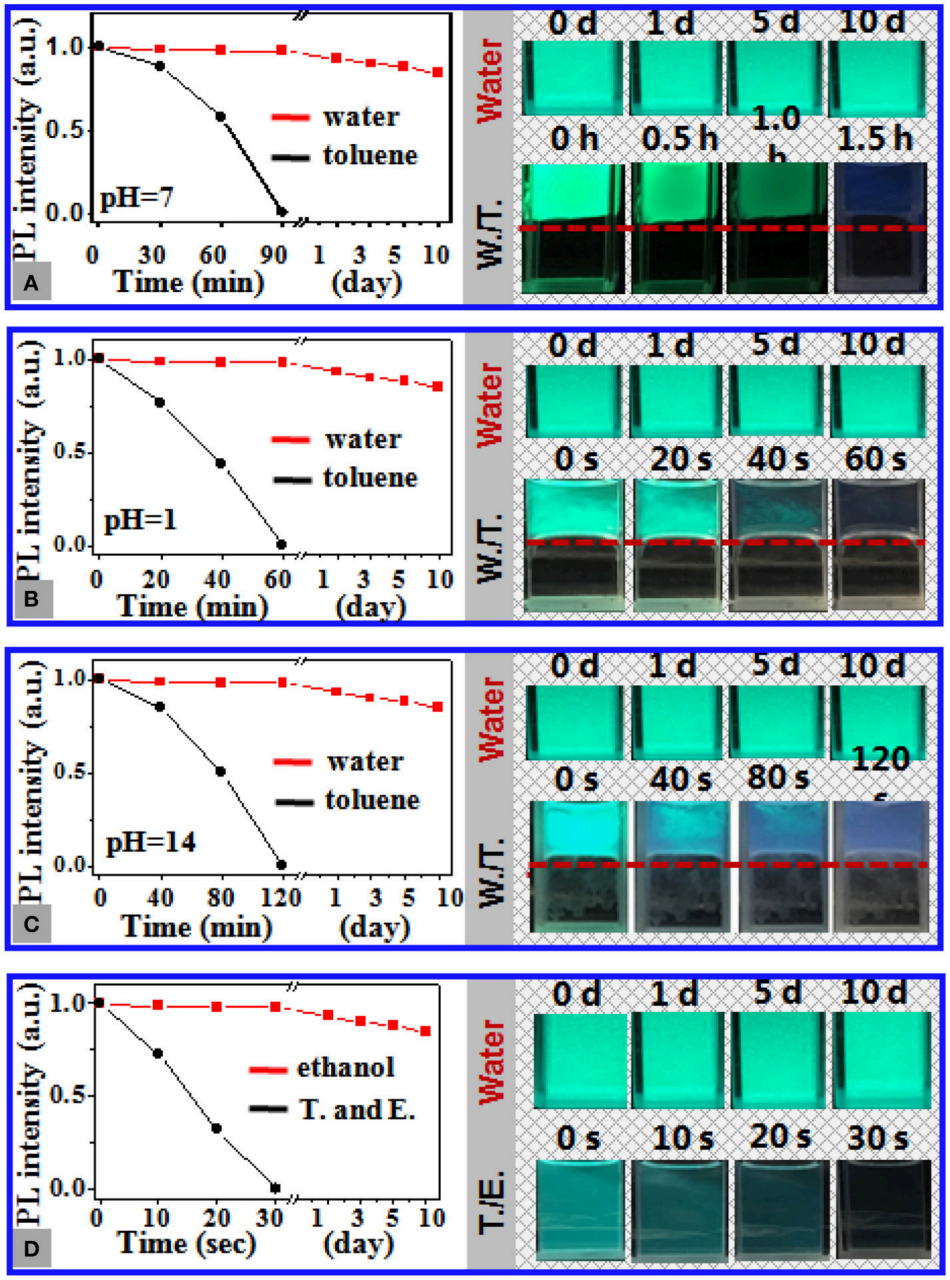
**(A–C)** Temporal evolution of the PL intensity of the dispersions of 11 nm CsPbBr_3_ NCs in toluene (black curves) and the aqueous dispersions of PS particles loaded with the same CsPbBr_3_ NCs at pH of **(A)** 7, **(B)** 1, and **(C)** 14 (red curves). The insets show the photos of the aqueous dispersions of the NC-loaded PS composite particles during storage at the corresponding pH and those of the toluene dispersions of the NCs placed in water at the corresponding pH, in which the toluene/water interfaces are highlighted by the red dashed lines. **(D)** Temporal evolution of the PL intensity of the toluene dispersion of the CsPbBr_3_ NCs (black curve) and the aqueous dispersions of the NC-loaded PS composite particles (red curve) in the presence of ethanol of the volume fraction of X vol. The photos of the corresponding dispersions are shown in the inset. The storage time is marked in the photos. The photos are shot under irradiation of UV light at 365 nm.

The stability of 11 nm CsPbBr_3_ NCs loaded in 1 μm PS particles in aqueous media was further assessed by monitoring the temporal evolution of the PL intensity of the composite particles at different pH values and in the presence of ethanol in water. Figure 2 shows that the PL of the NC dispersions in toluene suffered from rapid and severe deterioration upon being carefully placed in water, which was significantly expedited with the pH deviating from 7 or by adding ethanol into water (Figures 2B-D). The toluene dispersions of the NCs became slightly fluorescent after being in contact with neutral water (pH 7) for 1.5 h (Figure 2A). In contrast, minimal change in the PL of the NCs loaded in the PS particles was perceived after incubation of the composite particles in water for 10 days, regardless of the pH values and the presence of ethanol (Figure S7). The quantitative assessment revealed ~15% reduction in the PL intensity of the aqueous dispersions of the composite particles after 10 days, which, however, showed slight dependence on the pH value and the presence of ethanol in water. The excellent chemical stability of the NCs loaded in the PS particles against water was further validated by the fact that lead ions were slightly detectable in the surrounding aqueous media of the composite particles by means of inductively coupled atomic emission spectroscopy (Table S1).

**Figure 3:**
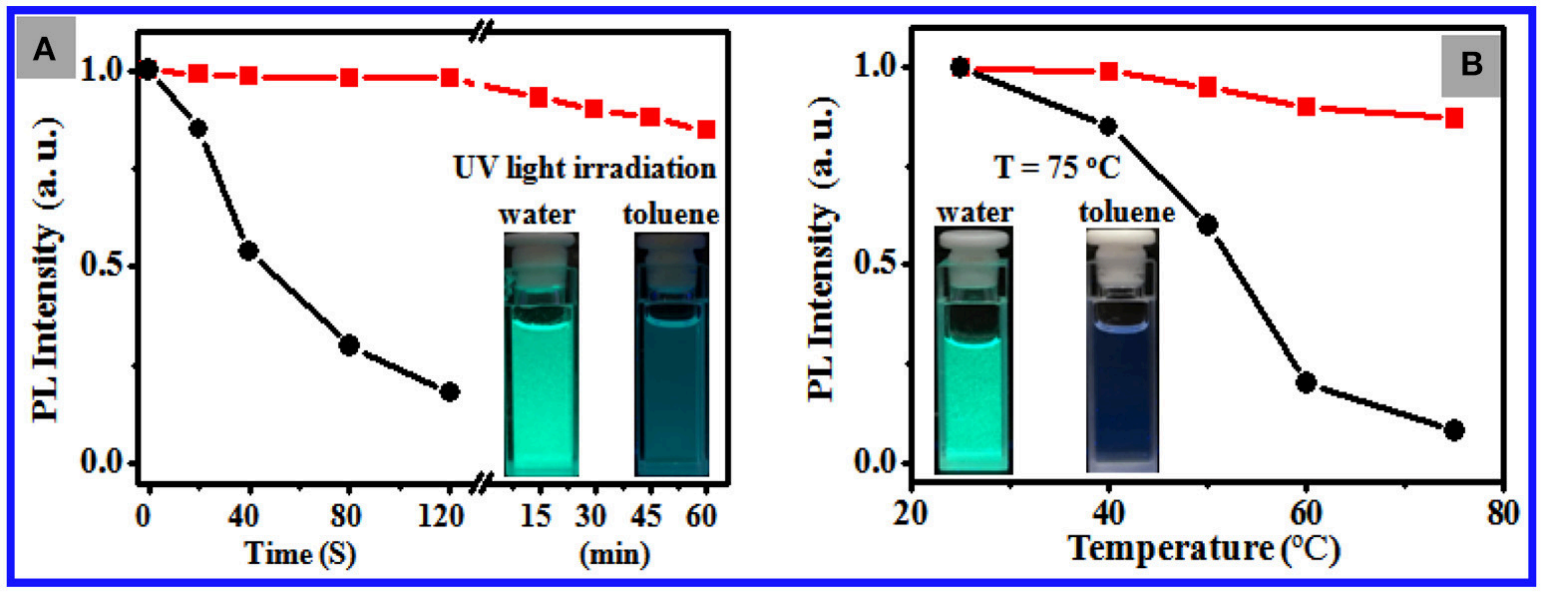
**(A)** Temporal evolution of the PL intensity of the toluene dispersions of 11 nm CsPbBr_3_ NCs (black curve) and the aqueous dispersion of the NC-loaded PS composite particles (red curve) under irradiation of light at 365 nm at a power of 8 W. The inset shows the photos of the corresponding composite particle dispersion in water after 30 min of irradiation (left panel) and the corresponding NC dispersions in toluene shot after 2 min of irradiation (right panel). **(B)** Plots of the PL intensity of the toluene dispersions of 11 nm CsPbBr_3_ NCs (black curve) and the aqueous dispersion of the NC-loaded PS composite particles (red curve) vs. environmental temperature. The inset shows the photos of the corresponding composite particle dispersion in water (left panel) and the corresponding NC dispersions in toluene after being warmed up to 75°C.

Figure 3A shows that 2 min of exposure to strong UV light at 365 nm results in 95% reduction of PL intensity of 11 nm CsPbBr_3_ NCs dispersed in toluene and ~6 nm blue shift as a result of photooxidation. By contrast, the NCs loaded in the PS particles retain 80% of their PL intensity after 30 min of irradiation, which indicated noticeably improved photostability (Figure S8). Figure 3B shows that the PL of the NCs dispersed in toluene significantly weakened with the increase in environmental temperature and was observed slightly at 75°C, whereas the NCs loaded in the PS particles in water maintained ~87% of the initial PL intensity at 75°C with minimal shift in emission position (Figure S9). Overall, these data endorse the excellent chemical and structural stability of the CsPbBr_3_ NCs loaded in the PS particles in water against pH, temperature, light, and polar organic solvents.

**Figure 4:**
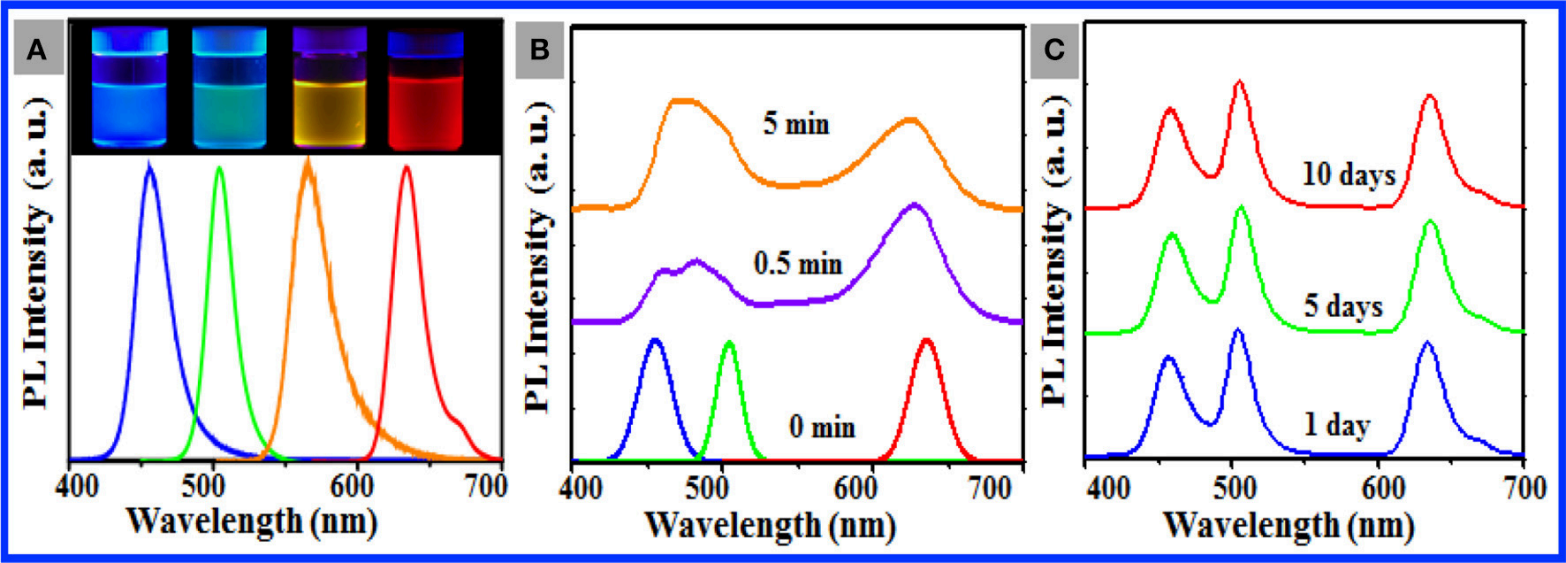
**(A)** PL spectra of the aqueous dispersions of as-prepared composite particles consisting of 1 nm PS particles loaded with 10 nm CsPb(Cl/Br)_3_ NCs (blue curve), 11 nm CsPbBr_3_ NCs (green curve), 12 nm CsPb(Br/I)_3_ NCs (orange curve), and 10 nm CsPbI_3_ NCs (red curve). The photos of the corresponding dispersions shot under UV irradiation are shown in the inset. **(B)** PL spectra of the toluene dispersions of the ternary mixtures of CsPb(Cl/Br)_3_ NCs, CsPbBr_3_ NCs, and CsPbI_3_ NCs recorded immediately after mixing for 30 s (purple curve) and incubated for 5 min under ambient condition (yellow line). For good comparison, the PL spectra (0 min) of these individual CsPb(Cl/Br)_3_ NCs (blue curve), CsPbBr_3_ NCs (green curve), and CsPbI_3_ NCs (red curve) are shown in **(B)**. **(C)** PL spectra of the aqueous dispersions of the ternary mixtures of the composite particles comprising CsPb(Cl/Br)_3_ NCs, CsPbBr_3_ NCs, and CsPbI_3_ NCs **(A)** during incubation under ambient condition for 1 day (blue curve), 5 days (green curve), and 10 days (red curve).

Our stepwise solvent exchange methodology slightly depends on the nature of the target perovskite NCs, which enables us to load different CsPbX_3_ NCs (Figure S10) into the PS particles [e.g., 10 nm CsPb(Cl/Br)_3_ NCs with blue emission, 11 nm CsPbBr_3_ NCs with green emission, 12 nm CsPb(Br/I)_3_ NCs with yellow emission, and 12 nm CsPbI_3_ NCs with red emission] to manufacture aqueous dispersions of various NC-loaded PS particles with single-emission colors (Figure 4A). The PL behavior of the resulting composite particles was fairly comparable with that of the original NCs (Figure S11), whereas the slight NC aggregation in the PS particles may account for a tiny red shift of 1–2 nm observed for the composite particles and the appearance of a tiny emission shoulder at 650 nm for the CsPbI_3_ NC-loaded particles (Figure 4A). As documented in the literature (Akkerman et al., 2015; Nedelcu et al., 2015), dynamic anion exchange occurs between perovskite NCs in multiple NC systems. This phenomenon is a serious technical shortcoming for the applicability of perovskite NCs in the field where multiple color display or luminescence is imperative. Our aforementioned success motivated us to intentionally mix the resulting composite particles loaded with 10 nm CsPb(Cl/Br)_3_ NCs, 11 nm CsPbBr_3_ NCs, and 12 nm CsPbI_3_ NCs in water to test the anion exchange between the loaded NCs. Figure 4B shows noticeable broadening of the PL spectra of the ternary NC mixtures of the CsPb(Cl/Br)_3_, CsPbBr_3_, and CsPbI_3_ NCs in toluene immediately upon mixing of these three QDs, with resulting noticeable spectral shifts of individual NCs. The PL spectra of the CsPb(Cl/Br)_3_ and CsPbBr_3_ NCs became slightly distinguishable after 5 min of incubation. In contrast, Figure 4C shows that after the three types of as-prepared composite particles were incubated in water for 5 days, the PL spectral profile of the individual particles remained slightly changed in the PL position and spectral profile, which indicated that minimal anion exchange occurred between the NCs loaded in the PS particles. This finding provides additional confirmation of the outstanding chemical stability of CsPbX_3_ NCs loaded in PS particles in aqueous media. It should be noted that the lead toxicity raises concerns for the use of perovskites in many situations. To determine if lead escaped from the as-prepared sample (purified three times by centrifugation and redispersion) in water within 10 days, inductively coupled atomic emission spectroscopy (ICP-AES) was conducted. No lead ions in water could be observed for the sample washed three times, indicating that the polymers not only protect the perovskite NCs from the environment but also effectively protect the environment from the toxic lead.

In summary, we succeeded in manufacturing water-borne CsPbX_3_ NC-embedded PS particles by directly incorporating prepared lipophilic perovskite NCs into the PS latex particles via solvent exchange with minimal alteration in the original structural and PL features of the NCs. Meanwhile, the hydrophilic surfaces of the PS host particles enabled excellent dispersion of the resulting composite particles in water. Their lipophilic dense interior matrices created excellent protection for vulnerable lipophilic perovskite NC guests against attack of acids, bases, and polar solvents, and also heat and light. This outstanding chemical and structural stability of the NCs loaded in the PS particles could minimize the release of toxic metallic ions (e.g., lead ions into aqueous media) and, more importantly, inhibit the anion exchange between the loaded NCs with different compositions to guarantee the color stability of the resulting composite particles. Overall, hydrophilic as-prepared perovskite NC-loaded PS particles would show promising prospects as innovative water-borne inks for printing of functional devices, such as wide color gamut LED backlight display. Furthermore, given that the present stepwise solvent exchange protocol is fairly trivial to operate and independent of the chemical nature of NC guests and latex hosts, it will enable us to adopt a broad spectrum of NCs and polymer particles to develop water-borne ink formulations in advanced inkjet printing.

## Author contributions

KH, MZ, and LP contributed to the qunatum dots synthesis. BL, DL, and QM contributed to the PS particles and imaging of particles. RX, DW, and WY contributed to the idea and writing.

### Conflict of interest statement

DL was employed by China Star Optoelectronics Technology Co. Ltd. The remaining authors declare that the research was conducted in the absence of any commercial or financial relationships that could be construed as a potential conflict of interest. The reviewer NG and handling Editor declared their shared affiliation.
