# Supplementary material for: Water-Borne Perovskite Quantum Dot-Loaded, Polystyrene Latex Ink
Source: Front Chem. 2018 Oct 23;6:453. doi: 10.3389/fchem.2018.00453 (PMC6206898; doi:10.3389/fchem.2018.00453)
Supplement: Supplementary file 1 [file Data_Sheet_1.DOC]

Supporting Information

**Water Borne, Perovskite Quantum Dot-Loaded, Polystyrene Latex Ink**

*Keke Huang, Lucheng Peng, Baijun Liu, Dongze Li, Qiang Ma,Mingyao Zhang, Renguo Xie, ^*^ Dayang Wang, and Wensheng Yang*

Dr. K. K. Huang, L. C. Peng, Dr. Q. Ma, Dr. Z. Shi, Prof. R. G. Xie, Prof. W. S. Yang

*State Key Laboratory of Inorganic Synthesis and Preparative Chemistry, College of Chemistry Jilin University, Changchun 130012, China*

*Dr. B. Liu, Dr. M. Zhang*

*Engineering Research Center of Synthetic Resin and Special Fiber, Ministry of Education,*

*Changchun University of Technology, Changchun, 130012, China,*

*Dr. D. Li*

*China Star Optoelectronics Technology Co. Ltd Shenzhen, 518132, China*

*E-mail: renguoxie@jlu.edu.cn*


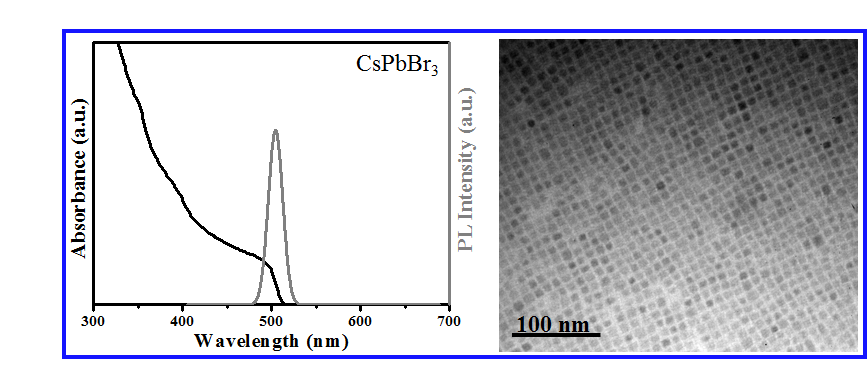


Figure S1. UV−vis absorption and fluorescence spectra of as-prepared 11 nm CsPbBr_3_ quantum dots (left). TEM image of the CsPbBr_3_ quantum dots (right).


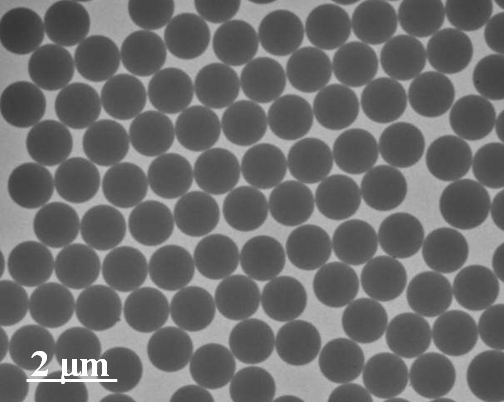


Figure S2. TEM image of polystyrene particles.


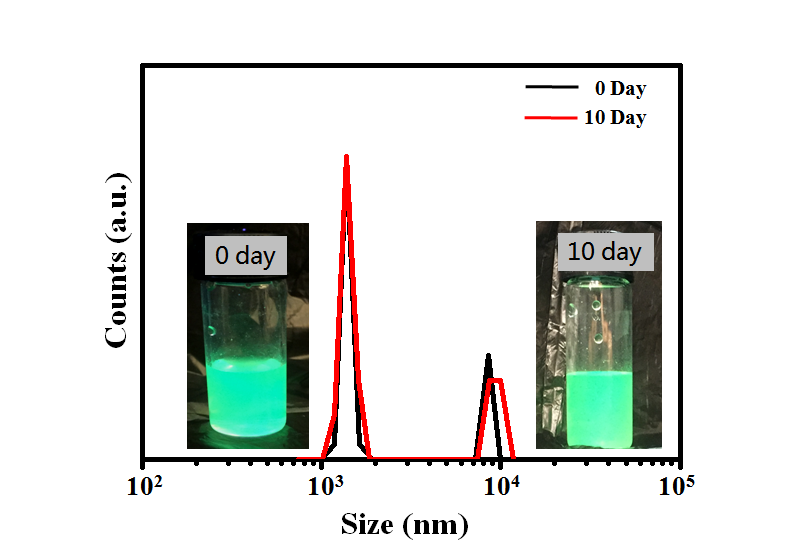


Figure S3. DLS measurements of QDs-PS composite at different time under UV light (λ = 365 nm); black line:0 day; red line: 10 days.


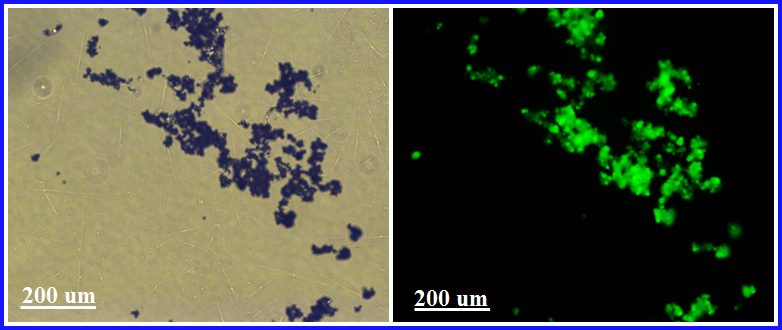


Figure S4. Fluorescence micrograph of the CsPbBr_3_ QDs loaded PS composite particles before (left) and after (right) UV irradiation (λ = 365 nm).


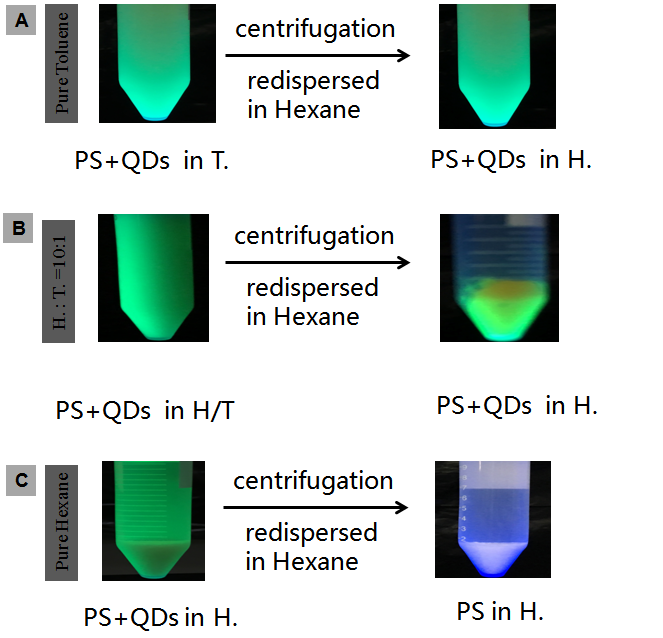


Figure S5. Digital pictures for incorporation of lipophilic perovskite QDs into PS particles under different ratios of hexane (H) v.s. toluene (T). (A) pure toluene; (B) T:H=1:10; (C) pure hexane. (λ = 365 nm)


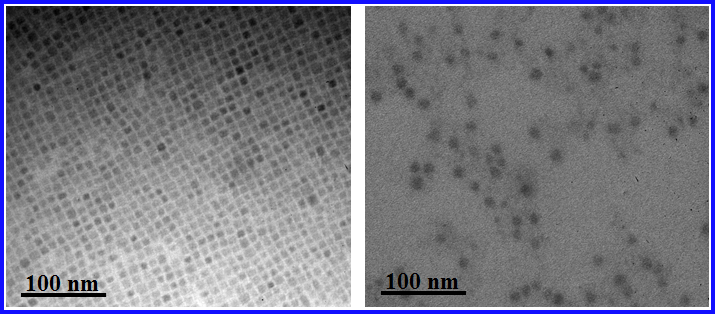


Figure S6. TEM images of QDs before (left) and after incorporation into PS particles (right).


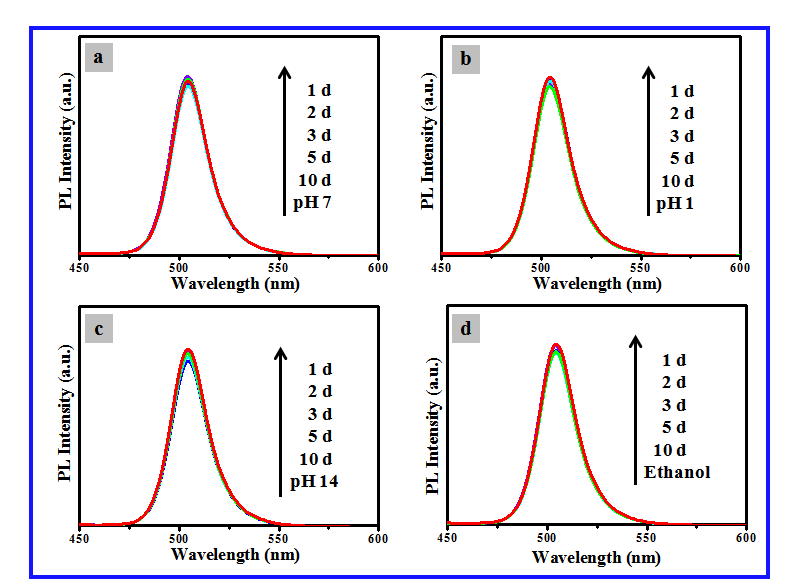


Figure S7. Temporal evolution of the fluorescence spectra of PSP loaded with perovskite QDs dispersed in water (a: pH=7; b: pH=1; c: pH=14) and ethanol (d), respectively. The storage time and pH values are given in the spectra.

Table S1. Pb ions in water determined by inductively coupled atomic emission spectroscopy (ICP-AES). X: no detectable Pb ions in water; О: Pb ions were detected in water.


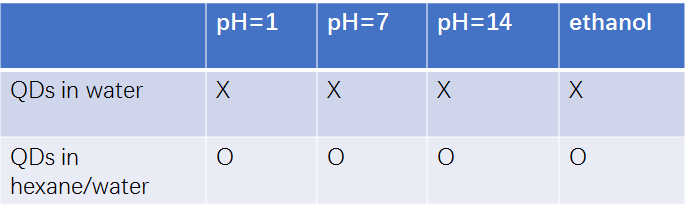


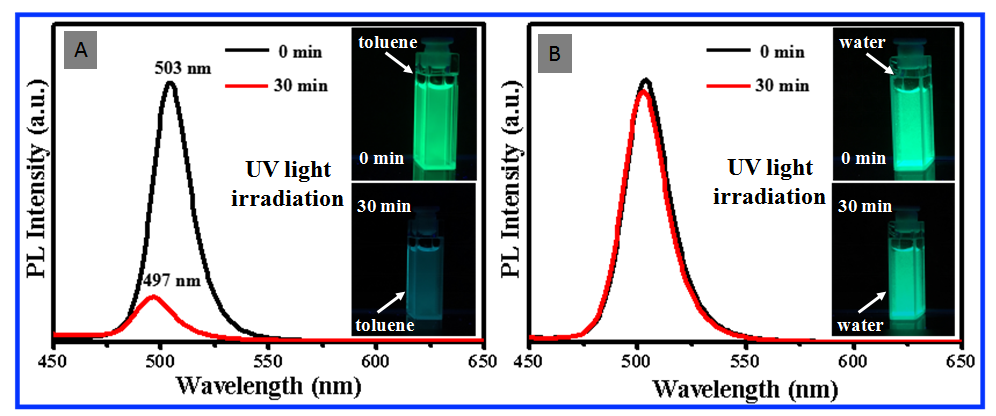


Figure S8.The fluorescence spectra of perovskite QDs in toluene (A) and water (B) before and after UV light irradiation 30 min. Inset:the pictures of the samples before and after UV light irradiation 30 min (the UV lamp, λ = 365 nm).


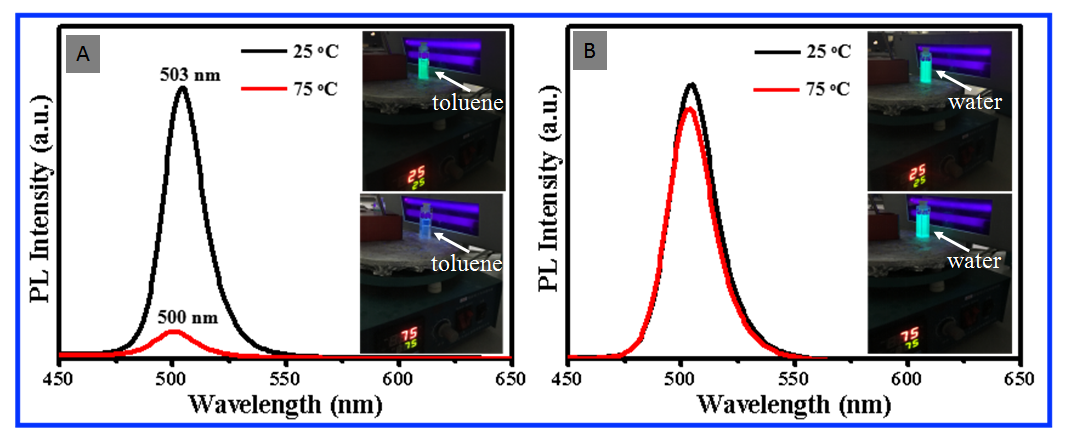


Figure S9. The fluorescence spectra of perovskite QDs in toluene (A) and water (B) at 25 and 75 ^o^C, respectively. Inset:the pictures of the samples at 25 and 75 ^o^C.


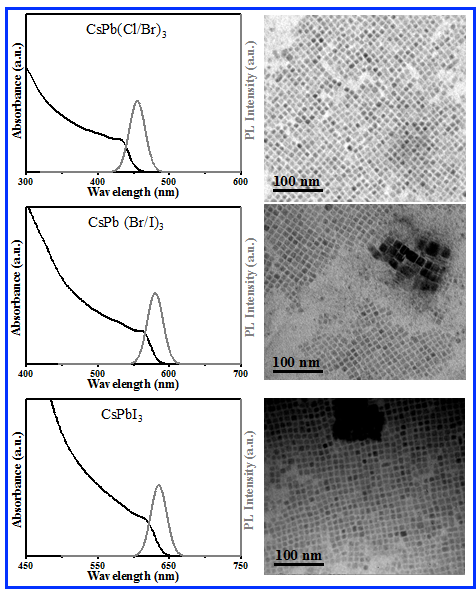


Figure S10. The UV−vis absorption and fluorescence spectrum (left) and Transmission electron microscopy images (right) of ∼8.4 nm CsPbX_3_ NCs after treatment with chloride and iodide anions.


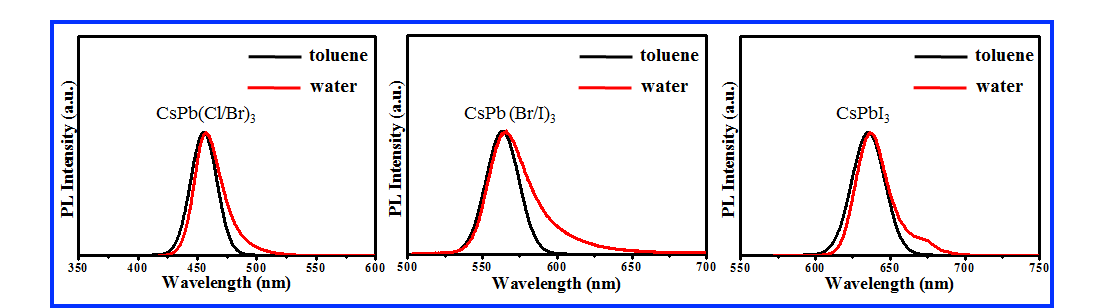


Figure S11.The fluorescence spectra of perovskite QDs dispersed in toluene (black lines) and QDs-PS particles in water (red lines).

Figure S12. TGA data for QDs loaded into PS particles at a constant heating rate of 5 °C min^−1^ under a flowing nitrogen atmosphere. The samples were dried in a vacuum oven overnight at 50 ^o^C with a reduced pressure (30 in. Hg) before analysis. The initial mass of QD loaded particles is 10.344 mg, the final mass of samples is 10.308 mg after 700 ^o^C.

Figure S13. TGA data for Pure PS particles at a constant heating rate of 5 °C min^−1^ under a flowing nitrogen atmosphere. The initial mass of QD loaded particles is 10.386 mg, the final mass of samples is almost zero after 700 ^o^C.
